# Differentiating Atypical BPPV from Central Positional Vertigo: A Narrative Review

**DOI:** 10.3390/neurosci7020032

**Published:** 2026-03-03

**Authors:** Giorgos Sideris, George Korres, Ilias Lazarou, Eleni Vasileiou, Amanda Male, Diego Kaski

**Affiliations:** 1 2nd Ear Nose Throat Department, Attikon University Hospital, 124 62 Athens, Greece; gfkorres@gmail.com (G.K.); hlias_laz@hotmail.com (I.L.); elenvasi7@yahoo.gr (E.V.); 2 SENSE Research Unit, Institute of Neurology, University College London, London WC1N 3BG, UK; a.male@nhs.net (A.M.); d.kaski@ucl.ac.uk (D.K.)

**Keywords:** BPPV, positional vertigo, central positional nystagmus, atypical BPPV, vestibular positional testing, cerebellar dysfunction

## Abstract

While typical benign paroxysmal positional vertigo (BPPV) presents with reproducible patterns of nystagmus and vertigo during positional testing, atypical variants often deviate from typical patterns, making diagnosis more complex. Recognizing atypical BPPV is crucial to avoid misdiagnosis and inappropriate management. This study aims to describe the clinical spectrum of atypical BPPV, differentiate it from central positional vertigo, and provide practical diagnostic guidance for clinicians. A narrative review was conducted to explore the clinical spectrum of atypical BPPV. Findings indicate that it may present with vertigo without nystagmus, conflicting torsional components in bilateral cases, or persistent symptoms despite repositioning maneuvers. Canal switch and pseudo-spontaneous nystagmus have also been described. Although these variants may mimic central etiologies, the absence of consistent neurological signs supports a peripheral mechanism. Diagnosis relies on detailed assessment of nystagmus characteristics—such as latency, /duration, and direction—as well as the exclusion of red flags, like direction-changing nystagmus without head movement, vomiting, or non-positional ocular motor abnormalities. Atypical BPPV remains a diagnostic challenge and requires careful bedside assessment and clinical testing. Understanding these variants is essential for timely and appropriate treatment. When doubt persists and resolution with treatment does not occur, neuroimaging should be considered to exclude central pathology.

## 1. Introduction

The term atypical positional vertigo describes cases where the nystagmus characteristics expected for benign paroxysmal positional vertigo (BPPV) are not present. Atypical forms of BPPV appear to be more prevalent than previously estimated, accounting for up to 40% of cases in tertiary referral hospitals [1]. Some studies suggest that BPPV may be more common with advancing age and in patients with comorbidities such as osteoporosis, and that older individuals may present more often with atypical symptoms and delayed diagnosis, underscoring the need for careful differentiation from central positional nystagmus (CPN) [2]. These variants are not rare in routine neuro-otological practice; however, their underlying pathophysiology remains incompletely understood and continues to be debated. In such cases, the cause may be a CPN or an atypical variant of BPPV. Differentiating between these entities is important because BPPV can be successfully treated with simple bedside maneuvers, whereas CPN will not respond to these and may require additional investigations to exclude potentially sinister causes [3]. Moreover, both typical and atypical BPPV, as well as central vestibular disorders—even when not caused by life-threatening conditions—can significantly impair quality of life [4].

BPPV is attributed to the displacement of otoconia within the semicircular canals, most commonly affecting the posterior canal, which is particularly susceptible due to its anatomical orientation and gravitational dependency within the inner ear [5]. Atypical forms of BPPV may involve multiple canals, uncommon triggering positions, or prolonged vertigo, all of which can contribute to diagnostic uncertainty [6]. In contrast, the presence of nystagmus during the Dix–Hallpike test that exhibits central features may point to underlying central nervous system pathology rather than a peripheral vestibular cause [3]. Specifically, nystagmus that is direction-changing, persistent, or non-fatiguing with repeated positioning raises suspicion for central etiologies such as multiple sclerosis, stroke, tumor, or other neurological conditions. In such cases, the observed nystagmus may deviate significantly from the classical patterns of BPPV [7].

BPPV is typically diagnosed through bedside positional testing—most notably the Dix–Hallpike test for posterior canal involvement, the supine roll test for horizontal canal involvement, whereas anterior canal BPPV is usually identified during the Dix–Hallpike or straight head-hanging maneuver by eliciting a characteristic downbeating torsional nystagmus. In typical BPPV, these manoeuvers provoke vertigo and induce nystagmus [8]. Diagnostic confirmation relies on specific characteristics of the elicited nystagmus: presence of latency, a crescendo–decrescendo pattern, short duration, and consistency with the plane of the affected canal (vertical, horizontal, or torsional) [8]. Despite attempts to develop objective diagnostic tests, BPPV remains primarily a clinical diagnosis, which may complicate the differential diagnosis in atypical presentations [5]. When nystagmus characteristics deviate from these expectations, the possibility of CPN—or a peripheral atypical variant—should be considered. The clinical question, therefore, becomes: how can these two entities be reliably differentiated?

This narrative review aims to synthesize current knowledge on atypical variants of BPPV, focusing on their clinical presentation, underlying mechanisms, and distinguishing features from central positional vertigo. Emphasis is placed on identifying diagnostic red flags and proposing a structured approach to improve diagnostic accuracy in challenging presentations of positional vertigo. Rather than providing an exhaustive description of specific therapeutic maneuvers for each subtype, the goal is to offer practical insights into diagnostic reasoning and management principles in complex cases.

## 2. Materials and Methods

We performed a narrative literature search to support this review on atypical BPPV and its differentiation from central positional vertigo and CPN. The search strategy included combinations of keywords related to positional vertigo, atypical BPPV variants, ocular motor findings, diagnostic bedside maneuvers, and central nervous system causes of positional dizziness. Relevant articles were identified through electronic databases, including PubMed (MEDLINE) and Scopus, covering sources from the earliest clinical descriptions of positional vertigo syndromes to recent advances in diagnostic interpretation and neuro-otological assessment. The search terms used included “benign paroxysmal positional vertigo”, “BPPV”, “atypical BPPV”, “refractory BPPV”, “positional vertigo”, “positional nystagmus”, “central positional vertigo”, “central positional nystagmus”, “CPN”, “downbeat nystagmus”, “direction-changing nystagmus”, “persistent nystagmus”, “apogeotropic nystagmus”, “cupulolithiasis”, “canalithiasis”, “Dix–Hallpike”, “supine roll test”, “video-oculography”, and “canalith repositioning manoeuvers”, including publications up to December 2025. Inclusion criteria were studies with direct clinical relevance to positional vertigo assessment, including publications describing nystagmus patterns, diagnostic criteria, differential diagnosis, and response to repositioning manoeuvers in peripheral and central etiologies. Exclusion criteria included studies not focused on positional vertigo/nystagmus, publications primarily addressing unrelated vestibular disorders without positional components, and articles lacking sufficient clinical data for diagnostic interpretation. Articles were selected based on their relevance to the clinical objective of distinguishing atypical peripheral positional syndromes from central causes, including evidence from observational studies, case series, clinical reviews, and consensus-based neurotology references. Data from each selected article were extracted regarding clinical presentation, positional testing findings, characteristics of nystagmus (direction, latency, duration, fatigability, and fixation suppression), diagnostic work-up (including indications for neuroimaging), and reported management approaches. The extracted information was then synthesized to construct a clinically oriented overview emphasizing key distinguishing features, diagnostic pitfalls, and practical bedside decision-making in patients presenting with atypical positional vertigo. This narrative approach was selected due to heterogeneity in study designs and variability in diagnostic criteria and terminology across the literature.

## 3. Results and Discussion

### 3.1. Mechanisms and Causes of CPN

CPN is a type of nystagmus elicited by changes in head position relative to gravity but caused by central (brain or brainstem) rather than peripheral (inner ear) pathology. The underlying mechanisms are complex and incompletely understood but likely involve disrupted central integration of vestibular inputs.

#### 3.1.1. Pathophysiology of CPN

Several pathophysiological theories have been proposed to explain the genesis of CPN. These mechanisms often implicate cerebellar or brainstem structures involved in vestibular signal processing and oculomotor control and include the following:

- Abnormal Central Processing/Integration: CPN is thought to arise from faulty central integration of signals from the semicircular canals (SCCs) and otolith organs, typically due to cerebellar or brainstem dysfunction. Particularly, prolonged velocity storage—a mechanism that extends vestibular responses to head rotation from the semicircular canals—is abnormally activated due to vestibulo-cerebellar dysfunction. This abnormality contributes to sustained or inappropriate nystagmus following head movement [3,9,10].
- Dysfunction of the Nodulus and Uvula: These cerebellar structures are key in processing vestibular information and maintaining spatial orientation. Their dysfunction may cause: (a) disinhibition of SCC signals—leading to an exaggerated post-acceleratory rebound phenomenon, where nystagmus persists after movement ceases [7,11,12]; and (b) a deficient rotational feedback mechanism—resulting in an inability to reconcile the discrepancy between predicted and actual gravity vectors, contributing to persistent nystagmus [13].
- Persistent Apogeotropic and Geotropic CPN: Geotropic nystagmus refers to nystagmus beating toward the ground (the lower ear) in a given head position, whereas apogeotropic nystagmus beats away from the ground (toward the upper ear). Apogeotropic CPN is associated with persistent erroneous estimation of gravity direction due to impaired function of the nodulus and uvula [14,15]. Geotropic CPN may result from cerebellar tonsil dysfunction, disrupting gravitational orientation and VOR modulation [15,16].
- Lesions in Other Brain Regions: Damage to fibers connecting the cerebellum (particularly the nodulus and uvula) to the vestibular nuclei, such as those passing through the inferior cerebellar peduncle, can result in similar nystagmus patterns. Such lesions are often seen in infarcts or tumors affecting posterior fossa structures [11,17,18].
- Mismatch in Eye Position Estimation: When the nystagmus direction is not aligned with head motion, a central mismatch between the eye position signal generated by the saccadic burst generator and the neural integrator may be implicated. This can be further exacerbated by altered otolith input [15,19].

The principal central anatomical structures implicated in the development of CPN are summarized in Table 1.

#### 3.1.2. An Overview of CPN Etiologies

CPN can arise from structural lesions of the posterior fossa that impair or disrupt the nodes involved in the above networks controlling head and eye coordination relative to changes in head position, or from non-structural causes that cause dysfunction in signal processing within these networks. This review is limited to describing some of the more common causes, acknowledging that this is by no means an exhaustive etiological list and that the underlying causes in many patients with non-structural CPN remain unknown.

- Vestibular Migraine (VM): VM is a central vestibular disorder that may present with spontaneous, positional, or motion-induced vertigo, and in some cases with positional nystagmus. It is unclear what the mechanism underlying positional nystagmus in VM is, but it has been argued that during acute migraine attacks, brainstem centers involved in pain and sensory modulation may hyperactivate the vestibular nuclei. Between attacks, compensatory suppression via the nodulus and uvula may contribute to abnormal vestibular processing and positional symptoms [20,21,22,23]. According to the Bárány Society criteria, the diagnosis of VM requires recurrent vestibular symptoms of moderate or severe intensity, a history of migraine, temporal association between vestibular and migraine features, and exclusion of alternative diagnoses [24]. Positional nystagmus is a common feature of acute VM [25,26,27,28,29]. The nystagmus characteristics include sustained oscillations (rather than brief paroxysms seen in canalolithiasis-type BPPV), low velocity, and a horizontal, vertical or torsional plane, or a combination of these. It is not unusual for the nystagmus to mimic that of BPPV, which makes the differential diagnosis more challenging and dependent on other clinical characteristics (e.g., vomiting, catamenial association, and headaches).
- Posterior Circulation Stroke: Ischemia in the vertebrobasilar system can impair the cerebellum, particularly the nodulus, uvula, or brainstem vestibular structures. This results in altered vestibular signal transmission and VOR dysfunction, often presenting with positional nystagmus without classic stroke symptoms [30,31,32,33]. In acute presentations, a comprehensive bedside assessment, including the HINTS+ examination (Head-Impulse, Nystagmus, Test-of-Skew, and hearing evaluation), is essential to help distinguish central causes from peripheral vestibular disorders.
- Cerebellopontine Angle and Fourth Ventricle Tumors: Lesions in the cerebellopontine angle or in the region of the fourth ventricle, even when small, may cause CPN. This can occur not only through alterations in cerebrospinal fluid (CSF) dynamics but also through direct compression or irritation of the vestibular nuclei located in the floor of the fourth ventricle and their cerebellar connections. Tumors such as vestibular schwannomas, ependymomas, medulloblastomas, or small astrocytomas may therefore produce positional nystagmus through mechanical distortion of these structures during head movements, even in the absence of overt hydrocephalus [34,35]. Clinically, these cases are often accompanied by marked nausea or vomiting and may also present with progressive unilateral auditory or vestibular symptoms, which may help distinguish them from BPPV.
- Multiple Sclerosis (MS): MS plaques within the brainstem can impair pathways connecting the vestibular nuclei, ocular motor nuclei, and somatosensory tracts. Demyelination in these areas may cause inappropriate vestibular signals and result in CPN [36,37,38,39].
- Other compressive lesions: Central compression of the vestibular nerve, especially by vascular loops or mass lesions, can cause episodic vertigo and nystagmus. This is often evident during positional maneuvers like the Dix–Hallpike or straight head-hanging and can be differentiated from BPPV, where the nystagmus characteristics include sustained (non-paroxysmal) nystagmus, pure vertical nystagmus, and a lack of response to repositioning maneuvers.

### 3.2. An Overview of Atypical Variants of BPPV

To contextualize the torsional rightbeating or torsional left-beating recognized features of atypical BPPV variants, the typical characteristics of posterior, horizontal, and anterior canal BPPV are outlined below:

(a) Posterior canal BPPV: In Posterior canal BPPV, the patient exhibits a positional geotropic torsional upbeat nystagmus in cases of canalolithiasis. It lasts from a few seconds up to a minute in the Dix–Hallpike position [40,41].
(b) Horizontal canal BPPV: In horizontal canal BPPV, the patient exhibits a positional horizontal nystagmus, which is apogeotropic in either cupulolithiasis or ampullary arm canalolithiasis and geotropic in non-ampullar arm canalolithiasis.
(c) Anterior canal BPPV: In this rare occasion, when the otoconia crystals are located in the anterior canal, the patient exhibits a vertical downbeat nystagmus with a torsional component that identifies the affected side [42].

In contrast to these well-characterized patterns, atypical variants of BPPV present with distinct clinical features that may complicate diagnosis and management.

#### 3.2.1. Bilateral BPPV

Bilateral BPPV involves the presence of BPPV in both ears, making diagnosis and treatment more challenging. It often occurs post-traumatically, e.g., after head injury, and can be identified by a positive Hallpike maneuver with upward and torsional nystagmus on both sides (geotropic). During the head-hanging maneuver, if only vertical (upbeating) nystagmus is observed, it could be an indication of bilateral posterior canal BPPV, as the torsional components from each side cancel each other out [43]. However, if there is a torsional component, it suggests pseudobilateral BPPV, usually due to inappropriate head positioning during testing. In cases of suspected bilateral BPPV, it is crucial to ensure the head is correctly oriented in the plane of the canal being explored to avoid misdiagnosis. Treatment involves repeated repositioning maneuvers over several sessions, and in refractory cases, referral for MRI brain imaging may be necessary to rule out any central cause [44]; however, evidence regarding the optimum number of treatment attempts is lacking.

#### 3.2.2. Multiple Canal BPPV on the Same Side

Multiple canal BPPV should be taken into consideration in patients after traumatic brain injury (TBI). Concurrent involvement of posterior and horizontal canals is the most common form [45], and in most mixed cases, the horizontal nystagmus is geotropic due to canalolithiasis, although cupulolithiasis has occasionally been reported. In such cases, treatment might be perplexing, and it should start with the position in which symptoms are most severe [46]. It is worth noting that patients following TBI may not report any dizziness or vertigo symptoms with position changes due to vestibular agnosia [47]. In these circumstances, assessing all patients following TBI for BPPV is recommended, and treating one canal at a time is advised.

#### 3.2.3. Vertigo Without Nystagmus on Dix–Hallpike

The most prevalent theory is that patients may have minimal otoconia particles stuck to the cupula or floating in the affected semicircular canal, enough to cause nausea and/or vertigo, but not enough to cause nystagmus [48]. A theory by Alvarenga et al. presents another explanation based on a change in the calcium metabolism and the consequent non-absorption of it. Calcium malabsorption would increase the quantity of small amounts of loose otoconia found in healthy individuals inside the semicircular canals enabling the triggering of vertigo upon head movement [49]. However, this proposition has not been validated elsewhere. This clinical presentation is also described as subjective BPPV, while some authors have described a similar entity as “Type 2 BPPV”, potentially representing the same phenomenon, particularly in patients evaluated outside the acute phase or with incomplete resolution and persistent postural symptoms [50,51].

#### 3.2.4. Sitting-Up Nystagmus and Vertigo (Scocco Variant)

A “sitting-up nystagmus and vertigo” variant has been described, where little or no nystagmus is elicited during the Dix–Hallpike test, but intense vertigo and characteristic posterior canal nystagmus occur upon returning to the sitting position. This presentation has been attributed to periampullary debris and potential canal narrowing that limits debris movement during the head-hanging phase, while debris displacement becomes more pronounced when the patient sits up. Management typically follows standard posterior canal liberatory maneuvers, while home-based exercises may be required in challenging cases [52].

#### 3.2.5. Dizziness with No Vertigo or Nystagmus in the Dix–Hallpike Position

A sensation of dizziness with no symptoms of vertigo or nystagmus is a common finding in vertigo clinics, since other central or peripheral pathologies (e.g, vestibular migraine or unilateral hypofunction) may cause a sensation of dizziness following any head movements in space [53]. Such dizziness is often triggered by any head movement, but cases have been reported in which the dizziness (without nystagmus and without a typical crescendo–decrescendo pattern of symptoms) occurs on one side only. In such instances, a diagnosis of BPPV is unlikely.

#### 3.2.6. Pseudo-Spontaneous Nystagmus on Lateral Canal BPPV

A patient with lateral canal BPPV, on the upright position, could exhibit signs of pseudo-spontaneous nystagmus, which beats in the same direction with the seated supine positioning nystagmus; both are consistent in identifying the affected side. The differential diagnosis between spontaneous nystagmus and pseudo-spontaneous nystagmus is easily achieved with the head pitch test in the sitting position: the pseudo-spontaneous nystagmus disappears when the head is bent forward 30° (neutral position); it reverses its direction with the head bent 60° forward; it becomes visible again when bringing the head in axis with the body; and it increases its intensity when extending the head about 30° backwards [54,55].

#### 3.2.7. Apogeotropic Nystagmus on Dix–Hallpike

One of the typical characteristics of posterior canal BPPV is geotropic torsional nystagmus. In rare cases, an apogeotropic variant could be present instead of a geotropic variant. The apogeotropic variant of posterior canal BPPV has been described in the literature and is thought to occur when otoconia are located in the non-ampullary arm or periampullary region of the canal, generating an inhibitory ampullipetal current and resulting in downbeating torsional nystagmus. Conversion into typical geotropic posterior canal BPPV during positional testing is considered a strong confirmatory sign, while clinical resolution after posterior canal liberatory maneuvers may also support the diagnosis [56,57,58,59]. Apogeotropic torsional nystagmus may be due either to anterior canal BPPV where it would need to be accompanied by a pure downbeating component, or cupulolithiasis of the posterior canal. This latter possibility can be determined by examining both sides on the Dix–Hallpike test. The anterior canal BPPV can be treated with the repositioning maneuver proposed by Yacovino, which does not require identification of the affected side, whereas apogeotropic posterior canal BPPV can be treated with the Quick Liberatory Rotation maneuver for typical posterior canal BPPV [60,61]. Careful evaluation of differences in intensity and direction of the nystagmus is fundamental. Identifying the triggering position or positions, observing nystagmus inversion when returning to the sitting position, and determining the affected canal and side help in selecting the most appropriate therapeutic maneuvers for each subtype [59].

#### 3.2.8. Yetiser Variant

Another atypical form of posterior canal BPPV, described by Yetiser, presents with a torsional–vertical nystagmus pattern that does not follow the expected canal physiology [55]. During positional testing, an initial response may suggest contralateral involvement, followed by features compatible with ipsilateral posterior canal stimulation. The proposed explanation involves debris near the common crus generating sequential endolymphatic flows, although this interpretation has been questioned due to inconsistencies with Ewald’s third law. Despite this debate, recognition of this variant underscores the need for careful analysis of torsional direction and nystagmus evolution during testing, as standard posterior canal maneuvers are usually effective.

#### 3.2.9. Short-Arm Canalolithiasis

Short-arm canalolithiasis is a posterior canal BPPV variant where otoconia are located on the utricular side of the cupula within the short-arm of the posterior canal. Clinically, this may produce subtle and more persistent downbeating nystagmus with ipsidirectional torsion at the end of the paroxysmal phase during the Dix–Hallpike test, differing from the transient torsional nystagmus seen in classic long-arm canalolithiasis. In some cases, nystagmus reversal may also be observed during liberatory maneuvers [62].

#### 3.2.10. Anatomical Variations in the Semicircular Canals and the Extraocular Muscles

The position of the semicircular canals in space might differ in some patients, therefore creating the need to modify the repositioning maneuvers accordingly. It has also been proposed that anatomical variation in extraocular muscle adhesion could be another cause of producing an atypical type of nystagmus, complicating the diagnosis and therapy [63].

#### 3.2.11. Heavy and Light Cupula

Heavy and light cupula occur when the cupula becomes relatively heavier or lighter than the surrounding endolymph, making it abnormally sensitive to gravity. Clinically, they cause prolonged positional nystagmus with little or no latency and may show direction-changing features with a “neutral point”. Their pathophysiology is not fully understood, and liberatory maneuvers are often less effective, except when cupulolithiasis is involved [64].

#### 3.2.12. Non-Resolving BPPV

A small number of patients never fully recover from seemingly typical BPPV, even after repeated manoeuvers. Canalith jam, where otoconia obstruct the narrowest portion of a semicircular canal, may also explain persistent symptoms and sustained nystagmus, most commonly in the horizontal canal but occasionally in the vertical canals as well [65,66,67]. In some other cases, a central but non-structural etiology may be the cause. In others, this may relate to variants in semicircular canal anatomy preventing otoliths from exiting the canal, but further research is necessary to explore this definitively. Post-traumatic BPPV can be associated with non-resolving symptoms, and vestibular rehabilitation exercises have been proven to be more helpful in refractory patients [68,69,70]. More research is required to explore treatment non-response.

#### 3.2.13. Canal Switch: A Possible Complication Following Epley’s Maneuver

A canal switch is not strictly an atypical variant of BPPV but rather a consequence of poor head position during attempted treatment. It occurs when otoliths, during or after the therapeutic maneuver, instead of moving into the utricle, migrate into another semicircular canal [46,71]. Reasons behind this may include patients with poor mobility or anatomical variations in the position of the semicircular canals in space. Another recognized mechanism of canal switch is performing a Dix–Hallpike maneuver too soon after a repositioning maneuver, potentially allowing partially mobilized otoconia to migrate into a different canal [72].

### 3.3. Key Differentiating Features: A Practical Approach

Differentiating peripheral positional vertigo (typically BPPV) from central positional vertigo/CPN relies mainly on careful interpretation of positional nystagmus behavior during bedside testing. In BPPV, nystagmus usually appears after a brief latency, is typically accompanied by intense vertigo, and follows canal-specific patterns such as torsional upbeating (posterior canal), horizontal geotropic or apogeotropic (horizontal canal), or downbeating with a torsional component (superior canal). Peripheral nystagmus is generally transient and fatigable, most often lasting less than one minute; however, in apogeotropic lateral canal BPPV, nystagmus may be prolonged, occasionally lasting several minutes, and may be associated with a limited paroxysmal response and show adaptation when maneuvers are repeated. In contrast, central positional nystagmus often presents with no latency, may occur with or without vertigo, and can show variable or non-canal-specific patterns. Central nystagmus is more likely to be persistent, may last several minutes, and typically shows limited fatigability or adaptation. Additionally, the typical reversal of nystagmus upon returning upright (often seen in BPPV after the Dix–Hallpike) is usually absent in central etiologies. When these atypical features are present—especially if symptoms do not improve after correctly performed maneuvers—central causes should be considered, and further evaluation may be warranted. For clinical guidance, Table 2 contrasts key features distinguishing peripheral from central causes of positional vertigo [8,73,74,75].

A crucial first step is the assessment for spontaneous nystagmus in the seated position, both with and without visual fixation. This is essential before proceeding to positional testing. Spontaneous nystagmus of either central or peripheral origin may be exacerbated by positional maneuvers—particularly the Dix–Hallpike test—likely due to top-down modulation of labyrinthine function via cerebellar and otolithic pathways [76]. Consequently, when spontaneous nystagmus is present, a diagnosis of BPPV should be made with caution and only when the positional nystagmus clearly differs in both plane and direction. This situation may occur, for example, in patients with BPPV following recent vestibular neuritis.

Further diagnostic refinement can be achieved by identifying additional signs suggestive of a central cause. These may be evident in the patient’s history or neurological examination and often involve posterior fossa dysfunction. Hallmarks include ocular motor abnormalities (e.g., broken smooth pursuit, hypermetric saccades, skew deviation, gaze-evoked nystagmus, or internuclear ophthalmoplegia), cranial nerve deficits (such as ocular motor palsies or facial weakness), or long tract signs (e.g., limb weakness or paraesthesia) [77]. Skew deviation deserves particular attention in this setting. It represents a vertical misalignment of the eyes due to involvement of central otolithic pathways within the brainstem or cerebellum, rather than dysfunction of the semicircular canals. As part of the ocular tilt reaction, it reflects disturbance of graviceptive processing in the posterior fossa. Clinically, skew deviation may be subtle and is best detected with an alternate cover test. When present in a patient with positional vertigo, it should raise strong suspicion of a central cause and prompt careful neurological evaluation. These central features, however, may be subtle or even absent, particularly if visual fixation is not suppressed during the examination. Notably, mild subjective vertigo should not reassure the clinician when ocular motor findings appear atypical. Video-oculography (VOG), when available, may further support bedside differentiation by improving detection of subtle torsional or vertical components and by allowing objective documentation of latency, duration, and direction of positional nystagmus. Importantly, VOG also facilitates examination with fixation removed, which can reveal nystagmus patterns that may otherwise appear reduced during direct observation. Although not universally available in all clinical settings, its use may increase diagnostic confidence in complex cases, particularly when positional findings are mixed, persistent, or non-canal-specific.

Despite traditional teaching, the characteristics of positional nystagmus alone may not reliably distinguish between central and peripheral causes, especially in the absence of overt central signs. Certain features, though, can be more specific for central pathology—for instance, a spontaneous change in the direction of nystagmus without any further head movement during testing (e.g., right-beating horizontal nystagmus on the right roll test that reverses to leftbeating while the head remains stationary).

In atypical or non-resolving cases of presumed BPPV, brain MRI is recommended [76,78,79]. However, a normal MRI does not rule out CPN, which remains a clinical diagnosis. Non-structural causes should still be considered. This highlights the value of careful bedside examination during positional testing. Follow-up and reassessment are also important in atypical positional vertigo, as diagnostic clarity may evolve over time. In typical BPPV, improvement is usually observed after appropriately performed repositioning manoeuvers, whereas persistence of the same atypical nystagmus characteristics across repeated assessments should prompt reconsideration of the diagnosis. Systematic documentation of positional findings at each visit, including provoking positions and nystagmus characteristics, may help distinguish true treatment failure from canal conversion, canal switch following repositioning maneuvers, or multicanal involvement, and support timely escalation when the expected clinical course of peripheral BPPV is not observed. This diagnostic challenge is amplified in atypical BPPV variants such as cupulolithiasis, bilateral involvement, multicanal disease, and canal conversion following manoeuvers, which may produce confusing positional nystagmus patterns and incomplete response to treatment.

**Figure 1:**
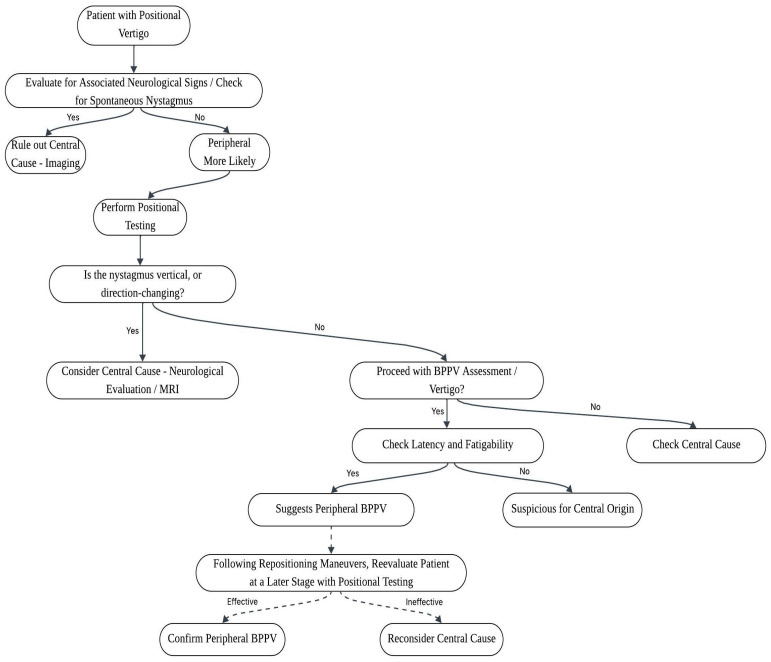
Diagnostic algorithm for patients presenting with positional vertigo.

Response to repositioning manoeuvers can also support the diagnostic process, since improvement after a correctly performed canalith repositioning manoeuver favors a peripheral mechanism, whereas repeated lack of improvement should prompt reconsideration of the diagnosis and evaluation for alternative causes. CPN should also be considered in the differential diagnosis, as it may be triggered by changes in head position relative to gravity and can closely mimic BPPV. Canalith repositioning maneuvers should be guided primarily by the observed nystagmus pattern rather than by symptoms alone, as accurate identification of the involved canal depends on careful interpretation of eye movement characteristics. In atypical or refractory cases, therapeutic success may improve when maneuvers employing different mechanical principles—gravitational, inertial, or mixed—are appropriately combined. Such an individualized approach is particularly relevant when debris location or canal dynamics are uncertain. CPN may present either paroxysmally or persistently and may appear as torsional downbeating nystagmus in the head-hanging position or as atypical apogeotropic patterns during Dix–Hallpike maneuvers, occasionally with overlap of both patterns. In clinical practice, CPN is often associated with additional central ocular motor signs, particularly cerebellar-related abnormalities, and it typically does not respond to liberatory maneuvers. For this reason, systematic follow-up and careful monitoring of response to repositioning treatment remain essential in atypical cases, helping to reduce the risk of misdiagnosis. Diagnostic confidence in a peripheral origin is strengthened when nystagmus converts into a typical canal-specific BPPV pattern during serial testing, whereas persistent atypical findings should prompt reassessment and consideration of central etiologies [50]. Given the overlap between atypical BPPV and central positional vertigo, a practical diagnostic approach should include a careful history focusing on onset pattern, triggers, recurrence, migraine features, and vascular risk factors, followed by positional testing with clear documentation of nystagmus direction, latency, duration, and fatigability, as well as evaluation of fixation suppression (ideally using video-oculography when available). Targeted canalith repositioning maneuvers should then be applied based on the suspected canal involvement, followed by structured follow-up reassessment to confirm resolution or detect canal conversion, with a low threshold for neuroimaging when findings remain inconsistent or when red flags are present. Ultimately, recognizing the limits of typical BPPV physiology is essential, as clinicians must balance avoiding unnecessary imaging against the risk of missing serious neurological disease; therefore, atypical positional nystagmus should not automatically be attributed to peripheral mechanisms, and early re-evaluation is warranted whenever the clinical course deviates from the expected response of BPPV. To support clinical decision-making and improve diagnostic accuracy in challenging cases of positional vertigo, Figure 1 illustrates a proposed diagnostic algorithm to aid in distinguishing typical from atypical BPPV and in identifying potential central causes of positional vertigo.

## 4. Conclusions

Atypical variants of BPPV, although less frequently encountered than classical posterior canal BPPV, represent a major diagnostic challenge because they may deviate from expected canal-specific nystagmus patterns and can closely overlap with central positional vertigo. Accurate identification requires not only familiarity with positional testing and careful interpretation of positional nystagmus characteristics but also an active effort to exclude central etiologies when findings are inconsistent, persistent, or atypical. In this setting, a high level of clinical vigilance is essential, particularly to reduce diagnostic delay and avoid repeated ineffective repositioning attempts in patients whose presentation does not align with peripheral vestibular physiology. In everyday clinical practice, increased awareness of atypical BPPV subtypes—such as multicanal involvement, bilateral disease, canal conversion, and cupulolithiasis—supports a more individualized diagnostic strategy and improves the likelihood of appropriate management. Importantly, patients with non-resolving positional vertigo or persistent positional nystagmus despite correctly performed therapeutic manoeuvers should be reassessed, as alternative diagnoses—including central causes—may need to be considered. In such cases, MRI should be considered to rule out posterior fossa and brainstem pathology, particularly in the presence of neurological signs, vascular risk factors, or atypical ocular motor findings. Future work should focus on improving the standardization of positional nystagmus interpretation and expanding access to objective eye-movement recording in routine practice.

## Figures and Tables

**Table 1:** Key Anatomical Structures Involved in Central Positional Nystagmus (CPN).

Anatomical Region	Structures Involved
Cerebellar Structures (Vestibulocerebellum)	Nodulus
	Uvula
	Cerebellar tonsils
	Flocculus
Brainstem Vestibular Structures	Vestibular nuclei (medial, lateral, superior, inferior)
	Inferior cerebellar peduncle
	Central otolithic pathways
Ocular Motor System	Ocular motor nuclei (III, IV, VI)
	Medial longitudinal fasciculus
	Neural integrator circuits
Posterior Fossa Structures	Cerebellum (nodulus/uvula region)
	Brainstem (pontomedullary junction)
	Cerebellopontine angle
	Fourth ventricle region

**Table 2:** Comparison of Νystagmus Characteristics in Peripheral versus Central Positional Vertigo.

Typical Characteristics of Nystagmus in BPPV	Central Positional Nystagmus
Latency	Usually no latency
Vertigo	With or without vertigo
Positional Geotropic torsional upbeat nystagmus (PSC) * Positional horizontal geotropic or apogeotropic nystagmus (HSC) ** Positional vertical downbeating nystagmus with a torsional component (SSC) ***	All variants
Fatigue (duration less than 1 min)	No fatigue, could last for minutes
Adaptation (when test is repeated shortly after initial testing)	Without adaptation
Reversal of nystagmus in the upright position following Dix–Hallpike	No reversal

* PSC: posterior semicircular canal, ** HSC: horizontal semicircular canal, *** SSC: superior semicircular canal.

## Data Availability

No new data were created or analyzed in this study. Data sharing is not applicable to this article.
